# Performance of classification and diagnostic criteria for IgG4-related disease and comparison of patients with and without IgG4-related disease

**DOI:** 10.1038/s41598-023-29645-2

**Published:** 2023-02-13

**Authors:** Masahiro Kogami, Yoshiyuki Abe, Taiki Ando, Ayako Makiyama, Ken Yamaji, Naoto Tamura

**Affiliations:** Department of Internal Medicine and Rheumatology, Juntendo University School of Medicine, 2-1-1 Hongo, Bunkyo-Ku, Tokyo, Japan

**Keywords:** Autoimmune diseases, Rheumatology

## Abstract

IgG4-related disease (IgG4-RD) was recently described in Japan. It is characterised by extensive organ involvement with tissue fibrosis. We assessed the performance of the 2019 American College of Rheumatology and European League Against Rheumatism (ACR/EULAR) classification criteria and the 2020 revised comprehensive diagnostic (RCD) criteria as well as differences between patients with and without IgG4-RD. In this retrospective, single-centre study of 50 patients admitted with suspected IgG4-RD, we evaluated the sensitivity and specificity of both criteria. We also compared clinical characteristics and laboratory data of patients with IgG4-RD (n = 42) and patients without IgG4-RD (n = 8). The ACR/EULAR classification criteria had 88.1% sensitivity and 87.5% specificity for IgG4-RD diagnosis. The RCD criteria had 100% sensitivity and 50% specificity. Patients with IgG4-RD had significantly more affected organs (p = 0.002). Patients with a single affected organ and IgG4-RD had significantly higher serum IgG4/IgG ratios (p = 0.027), lower serum C-reactive protein levels (p = 0.020), and lower total haemolytic complement activity (p = 0.044) than those without IgG4-RD. The ACR/EULAR classification criteria have high specificity and the RCD criteria have high sensitivity for diagnosing IgG4-RD. The number of affected organs is important for diagnosing IgG4-RD.

## Introduction

Immunoglobulin G (IgG) 4-related disease (IgG4-RD) was first described in Japan. It is characterised by extensive organ involvement with tissue fibrosis and IgG4-positive plasma cell infiltration^1^. Although the Japanese IgG4-RD team established comprehensive diagnostic (CD) criteria for IgG4-RD in 2011^2^, several issues with the 2011 CD criteria have been raised, such as difficulty in performing tissue biopsies in some patients with suspected IgG4-RD and concerns about the usefulness of serum IgG4 levels for diagnosis^3,4^. Because IgG4-RD and other diseases have overlapping clinical features, it is important to distinguish between them accurately. It is well known that some patients without IgG4-RD have high serum IgG4 levels and more IgG4-positive cells in affected tissues^5–7^.

In 2019, the American College of Rheumatology (ACR) and European League Against Rheumatism (EULAR) proposed criteria that can classify IgG4-RD with high specificity, without tissue biopsy^8^. However, there is concern about the exclusion criteria; patients positive for disease-specific antibodies are excluded from analysis for IgG4-RD before they are assessed for other autoimmune diseases. The revised comprehensive diagnostic (RCD) criteria for IgG4-RD were proposed by a team from the Ministry of Health, Labour and Welfare of Japan in 2020^9^. The 2020 RCD criteria were a revision of the 2011 CD criteria. Definite IgG4-RD, which includes patients who meet the organ-specific criteria for IgG4-RD and pathological diagnoses including storiform fibrosis and obliterative phlebitis, was added. However, there are still few studies about the performance of the 2020 RCD criteria in diagnosing IgG4-RD.

In this study, we evaluated the performance of both the 2019 ACR/EULAR classification criteria and the 2020 RCD criteria in diagnosing IgG4-RD. We also evaluated clinical differences between IgG4-RD and other diseases.

## Methods

### Patients

We retrospectively evaluated patients who were admitted to Juntendo University Hospital with suspected IgG4-RD from April 2010 to March 2022. Rheumatologists certified by the Japan College of Rheumatology diagnosed IgG4-RD based on the 2011 CD criteria^2^ or respective organ-specific criteria for IgG4-RD^10–16^ after excluding other diseases. We also did not include patients who were initially diagnosed with IgG4-RD but ultimately diagnosed with another disease. This study was approved by the ethics committee of Juntendo University Hospital (approval number E22-0363) and conducted in accordance with the principles of the Declaration of Helsinki. The need to obtain informed consent was waived by the ethics committee of Juntendo University Hospital because of the retrospective design.

### Clinical evaluation

We collected clinical data, including patient demographics, laboratory data at diagnosis, and outcomes, from medical records. The number of involved organs was counted from 11 lesions, based on a previous study^17^. The sensitivity and specificity of the 2019 ACR/EULAR classification criteria and the 2020 RCD criteria were evaluated by rheumatologists.

The 2019 ACR/EULAR classification criteria consist of a three-step process. Step 1 involved the entry criteria; a patient met the entry criteria if there were characteristic clinical or radiologic involvement in a typical organ. Step 2 involved the exclusion criteria; a patient met the exclusion criteria if they had any clinical, serologic, or pathologic exclusion items. If the patient met entry criteria and did not meet any exclusion criteria, Step 3 was initiated. Step 3 involved the inclusion criteria; a patient met the classification criteria for IgG4-RD if the total number of points over 8 domains was ≥ 20.

The 2020 RCD criteria contained three items: (1) clinical and radiological features, (2) serological diagnosis, and (3) pathological diagnosis. Patients who fulfilled all three items were diagnosed with definite IgG4-RD. Those who fulfilled items (1) and (3) were diagnosed with probable IgG4-RD. Those who fulfilled items (1) and (2) were diagnosed with possible IgG4-RD. Patients with a diagnosis of possible or probable IgG4-RD based on the CD criteria who fulfilled organ-specific criteria for IgG4-RD^10–16^ were also regarded as having definite IgG4-RD. In addition to definite IgG4-RD, probable IgG4-RD and possible IgG4-RD also met the 2020 RCD criteria. The details of both criteria were provided in previous publications^8,9^.

### Statistical analysis

Statistical analysis was performed using SPSS version 23.0 software (IBM Corp.; Armonk, NY). We used the Mann–Whitney *U* test to analyse continuous variables and Fisher’s exact test to evaluate categorical variables. Sensitivity referred to the proportion of patients with IgG4-RD who met the diagnostic criteria. Specificity referred to the proportion of patients with other diseases who did not meet the diagnostic criteria. The Kappa coefficient was used for assessing agreement between the criteria (> 0.80 was considered almost perfect reliability, 0.61–0.80 substantial, 0.41–0.60 moderate, 0.21–0.40 fair, 0.00–0.20 slight, and < 0.00 poor)^18^. To evaluate the predictive performance of clinical data, receiver operating characteristic (ROC) analysis was performed. We calculated the cut-off point using Youden’s index. *P*-values < 0.05 were considered statistically significant.

## Results

### Diagnostic performance of both criteria

A total of 50 patients with suspected IgG4-RD were admitted to our hospital and we diagnosed 42 patients with IgG4-RD. Patients who were not diagnosed with IgG4-RD had multicentric Castleman disease (n = 2), non–IgG4-related idiopathic retroperitoneal fibrosis (n = 2), malignant lymphoma (n = 1), dedifferentiated liposarcoma (n = 1), urothelial carcinoma (n = 1), and undiagnosed disease (n = 1). The ACR/EULAR classification criteria had 88.1% sensitivity and 87.5% specificity for IgG4-RD diagnosis. On the other hand, the 2020 RCD criteria had 100% sensitivity and 50% specificity for IgG4-RD diagnosis. There were 21 patients diagnosed with definite IgG4-RD, 1 patient with probable IgG4-RD, and 20 patients with possible IgG4-RD. The kappa coefficient for the criteria was 0.43.

### Clinical data of patients with versus without IgG4-RD

**Figure 1:**
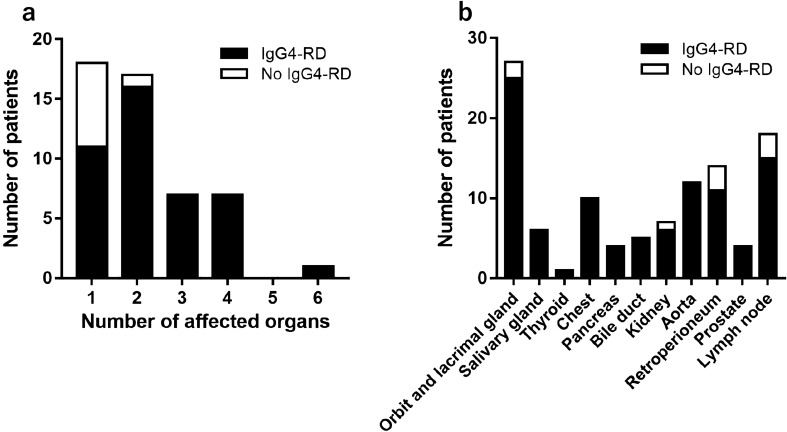
Affected organs by IgG4-RD status. (**a**) Number of patients by the number of affected organs. (**b**) Number of patients by affected organ. *IgG4-RD* immunoglobulin G4-related disease.

**Figure 2:**
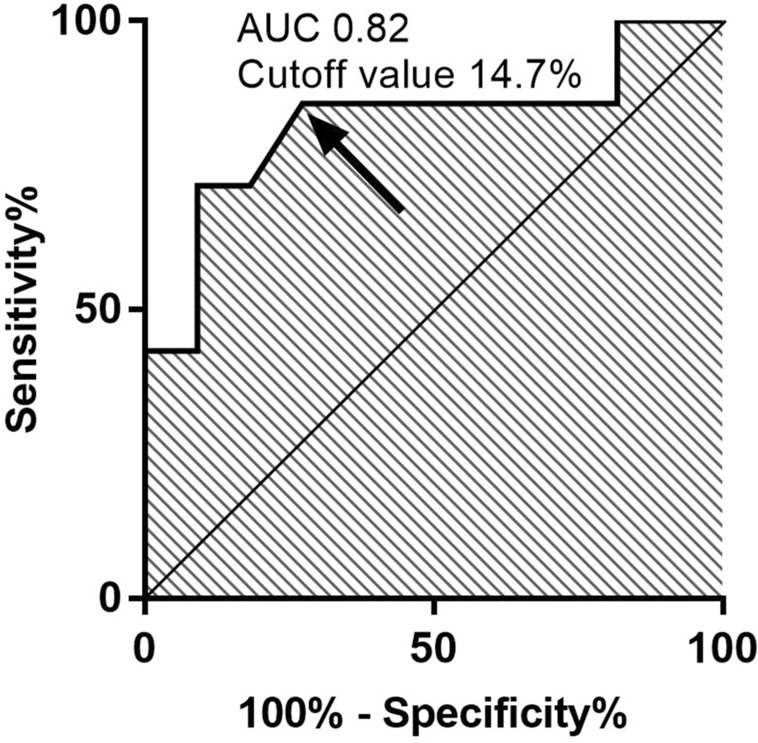
ROC curve for defining the optimal cut-off IgG4/IgG ratio in patients with a single affected organ. The ROC curve for distinguishing IgG4-RD from other diseases in patients with a single organ lesion. The cut-off value was 14.7%, AUC was 0.82, sensitivity was 90.9%, and specificity was 71.4%. *AUC* area under the curve, *IgG4-RD* immunoglobulin G4-related disease, *ROC* receiver operating characteristic.

We compared the clinical characteristics and laboratory data at admission of patients with versus without IgG4-RD. Only the number of affected organs differed significantly between these two groups (Table 1). Figure 1a shows the number of patients by the number of affected organs. Figure 1b shows the number of patients for each affected organ. In addition, we compared the clinical data of patients with versus without IgG4-RD who had a single affected organ (Table 2). Among patients with only a single affected organ, patients with IgG4-RD had significantly lower serum C-reactive protein (CRP) levels (median 0.10 mg/dL vs 0.31 mg/dL; p = 0.020), higher serum IgG4/IgG ratios (22% vs 12%; p = 0.027), and lower total haemolytic complement activity (CH50) (38.0 U/mL vs 48.9 U/mL; p = 0.044) than patients without IgG4-RD. When we examined ROC curves for serum IgG4/IgG ratio in patients with a single affected organ, we obtained an optimal cutoff value of 14.7% for differentiating between patients with versus without IgG4-RD (Fig. 2).

**Table 1:** Characteristics of patients with versus without IgG4-RD.

	IgG4-RD	No IgG4-RD	*P*
	n = 42	n = 8	
Characteristic			
Age, years, median (IQR)	68 (57–76)	72 (61–78)	0.59
Sex, female, n (%)	12 (28.6)	5 (62.5)	0.076
Affected organ			
Orbit and lacrimal gland, n (%)	25 (59.5)	2 (25.0)	0.079
Salivary gland, n (%)	6 (14.3)	0 (0)	0.33
Thyroid, n (%)	1 (2.4)	0 (0)	0.84
Chest, n (%)	10 (23.8)	0 (0)	0.14
Pancreas, n (%)	4 (9.5)	0 (0)	0.49
Bile duct, n (%)	5 (11.9)	0 (0)	0.40
Kidney, n (%)	6 (14.3)	1 (12.5)	0.69
Aorta, n (%)	12 (28.6)	0 (0)	0.091
Retroperitoneum, n (%)	11 (26.2)	3 (37.5)	0.40
Prostate, n (%)	4 (9.5)	0 (0)	0.49
Lymph node, n (%)	15 (35.7)	3 (37.5)	0.61
Multiple affected organs, n (%)	31 (73.8)	1 (12.5)	0.002*
Number of affected organs, median (IQR)	2 (1–3)	1 (1–1)	0.002*
Laboratory tests			
WBC, /μL, median (IQR)	5800 (4875–6625)	6250 (4900–8775)	0.43
Eosinophils, /μL, median (IQR)	269 (151–473)	271 (171–704)	0.63
Haemoglobin, g/dL, median (IQR)	12.8 (11.3–13.8)	11.7 (9.4–13.8)	0.32
Platelets, 10^4^/μL, median (IQR)	24.6 (19.1–26.7)	29.7 (21.0–41.2)	0.12
LDH, U/L, median (IQR)	149 (132–179)	183 (105–217)	0.81
Creatinine, mg/dL, median (IQR)	0.87 (0.66–1.17)	0.93 (0.60–1.19)	0.85
Ferritin, ng/dL, median (IQR)	173 (70–345)	117 (101–346)	0.95
CRP, mg/dL, median (IQR)	0.22 (0.10–0.45)	0.26 (0.08–5.9)	0.49
IgG, mg/dL, median (IQR)	2097 (1585–3293)	2196 (1544–4994)	0.87
IgG4, mg/dL, median (IQR)	427 (215–1500)	507 (54–1130)	0.32
IgG4 ≥ 135 mg/dL, n (%)	40 (95.2)	6 (75.0)	0.12
IgG4/IgG ratio, %, median (IQR)	24 (14–38)	14 (3–29)	0.061
IgA, mg/dL, median (IQR)	172 (120–224)	363 (151–460)	0.061
IgM, mg/dL, median (IQR)	59 (37–92)	66 (42–171)	0.58
IgE, U/mL, median (IQR)	319 (165–590)	289 (51–652)	0.95
CH50, CH50/mL, median (IQR)	39.6 (32.4–50.9)	48.9 (39.1–53.3)	0.099
C3, mg/dL, median (IQR)	89 (71–101)	99 (75–119)	0.23
C4, mg/dL, median (IQR)	17 (11–25)	20 (15–27)	0.074
sIL-2R, U/mL, median (IQR)	913 (513–1360)	865 (756–1750)	0.76

*P* values were determined using the Mann–Whitney *U* test or Fisher’s exact test, as appropriate.

*P < 0.05.

*IgG4-RD* immunoglobulin G4-related disease, *IQR* interquartile range, *WBC* white blood cells, *LDH* lactate dehydrogenase, *CRP* C-reactive protein, *Ig* immunoglobulin, *CH50* total haemolytic complement activity, *sIL-2R* soluble interleukin-2 receptor.

**Table 2:** Characteristics of patients with a single affected organ by IgG4-RD status.

	IgG4-RD with a single affected organ	Other disease with a single affected organ	*P*
	n = 11	n = 7	
Characteristic			
Age, year, median (IQR)	69 (52–76)	77 (60–78)	0.43
Sex, female, n (%)	5 (45.5)	4 (57.1)	0.50
Affected organ			
Orbit and lacrimal gland, n (%)	7 (63.6)	1 (14.3)	0.057
Salivary gland, n (%)	0 (0)	0 (0)	N/A
Thyroid, n (%)	0 (0)	0 (0)	N/A
Chest, n (%)	1 (9.1)	0 (0)	0.61
Pancreas, n (%)	0 (0)	0 (0)	N/A
Bile duct, n (%)	0 (0)	0 (0)	N/A
Kidney, n (%)	0 (0)	1 (14.3)	0.39
Aorta, n (%)	1 (9.1)	0 (0)	0.61
Retroperitoneum, n (%)	2 (18.2)	3 (42.9)	0.27
Prostate, n (%)	0 (0)	0 (0)	N/A
Lymph node, n (%)	0 (0)	2 (28.6)	0.14
Laboratory tests			
WBC, /μL, median (IQR)	5400 (4800–6400)	6000 (4700–6900)	0.48
Eosinophils, /μL, median (IQR)	178 (151–258)	196 (168–562)	0.29
Haemoglobin, g/dL, median (IQR)	12.7 (12.2–13.3)	11.2 (9.1–13.4)	0.18
Platelets, 10^4^/μL, median (IQR)	26.4 (22.3–29.9)	30.4 (22.3–43.7)	0.13
LDH, U/L, median (IQR)	161 (134–207)	194 (105–221)	0.93
Creatinine, mg/dL, median (IQR)	0.79 (0.65–0.93)	0.94 (0.60–1.22)	0.33
Ferritin, ng/dL, median (IQR)	70 (53–388)	117 (101–346)	0.48
CRP, mg/dL, median (IQR)	0.10 (0.03–0.20)	0.31 (0.14–7.1)	0.020*
IgG, mg/dL, median (IQR)	1562 (1379–1938)	1910 (1472–4996)	0.29
IgG4, mg/dL, median (IQR)	389 (216–433)	172 (54–721)	0.79
IgG4 ≥ 135 mg/dL, n (%)	10 (90.9)	5 (71.4)	0.33
IgG4/IgG ratio, %, median (IQR)	22 (16–25)	12 (3–16)	0.027*
IgA, mg/dL, median (IQR)	177 (145–256)	363 (190–460)	0.069
IgM, mg/dL, median (IQR)	110 (37–133)	66 (44–171)	0.66
IgE, U/mL, median (IQR)	359 (214–676)	557 (151–747)	0.86
CH50, U/mL, median (IQR)	38.0 (35.9–45.9)	48.9 (39.1–53.3)	0.044*
C3, mg/dL, median (IQR)	89 (82–93)	99 (75–119)	0.15
C4, mg/dL, median (IQR)	18 (17–22)	20 (15–32)	0.48
sIL-2R, U/mL, median (IQR)	533 (380–1180)	865 (529–1750)	0.15

*P* values were determined using the Mann–Whitney *U* test or Fisher’s exact test, as appropriate.

*P < 0.05.

*IgG4-RD* immunoglobulin G4-related disease, *IQR* interquartile range, *WBC* white blood cells, *LDH* lactate dehydrogenase, *CRP* C-reactive protein, *Ig* immunoglobulin, *CH50* total haemolytic complement activity, *sIL-2R* soluble interleukin-2 receptor.

## Discussion

In this study, we evaluated the performance of the classification and diagnostic criteria for IgG4-RD in patients admitted to hospital with suspected IgG4-RD. We confirmed that the 2019 ACR/EULAR classification criteria enabled disease classification with high specificity, similar to the results of a previous study^8^. We also found that the 2020 RCD criteria enabled diagnosis with high sensitivity. The kappa coefficient between the 2011 CD criteria and the 2019 ACR/EULAR classification criteria showed poor reliability in a previous study^19^; however, this study showed moderate reliability between the 2020 RCD criteria and 2019 ACR/EULAR classification criteria. One possible explanation is that the 2020 RCD criteria allowed for the diagnosis of definite IgG4-RD with organ-specific findings or additional pathological findings, which allowed for the diagnosis to be made regardless of serum IgG4 levels, thereby increasing the agreement between the two sets of criteria.

There were no significant differences in patients with versus without IgG4-RD in this study, other than in the number of affected organs. Serum IgG4 levels and IgG4/IgG ratio were considered useful for diagnosing IgG4-RD^20^ according to one study, but another study found them to be less useful^4^. These results also showed that it was difficult to differentiate IgG4-RD from other diseases based serum IgG4 levels and the IgG4/IgG ratio. However, we confirmed the usefulness of the IgG4/IgG ratio in a sub-analysis limited to patients with a single affected organ. Previous studies showed that many patients with IgG4-RD have multiple affected organs^21^ and as the number of affected organs increases, so do serum IgG4 levels^22^. However, patients with IgG4-RD who have relatively small and restricted lesions have been reported to have higher IgG4/IgG ratios, even in the absence of elevated serum IgG levels^20^. CH50 and serum CRP levels were also significantly lower in patients with IgG4-RD than patients without IgG4-RD among patients with a single affected organ. A previous study reported hypocomplementemia and low serum CRP levels in IgG4-RD^23^; we had similar results in this study. The mechanism underlying hypocomplementemia in IgG4-RD remains unclear, but it has been reported that IgG4-RD with hypocomplementemia is associated with more kidney or lung involvement and relapse^17^. Since serum CRP levels are often elevated in mimickers such as multicentric Castleman disease^5^, we might have identified a significant difference in serum CRP levels among patients with versus without IgG4-RD with a single affected organ. For the diagnosis of IgG4-RD, focusing on laboratory data such as the serum IgG4/IgG ratio relative to the number of affected organs is expected to help increase diagnostic accuracy.

This study has several limitations. It used a single-centre, retrospective design. Interpreting results for classification criteria may require caution, because the purpose of classification criteria is to create homogenous cohorts for clinical research and not for diagnosis. Due to the limited number of patients, multivariate analysis was not performed. The results of this study might need to be confirmed in larger studies.

In conclusion, the 2019 ACR/EULAR classification criteria have high specificity and the 2020 RCD criteria have high sensitivity for diagnosing IgG4-RD. The number of affected organs is also important for diagnosing IgG4-RD, with particular attention to the serum IgG4/IgG ratio in the case of single organ involvement.

## Data Availability

The data underlying this article will be shared upon reasonable request to the corresponding author.
